# Facile Fabrication of Zeolitic Imidazolate Framework-8@Regenerated Cellulose Nanofibrous Membranes for Effective Adsorption of Tetracycline Hydrochloride

**DOI:** 10.3390/molecules29174146

**Published:** 2024-08-31

**Authors:** Zhirong Wang, Qiuxia Fu, Dandan Xie, Fujie Wang, Guangyu Zhang, Haoru Shan

**Affiliations:** 1 School of Textile and Clothing, Nantong University, Nantong 226019, China; 15035184638@163.com (Z.W.); xiedd127@163.com (D.X.); w1651119684@163.com (F.W.); zgyu85@ntu.edu.cn (G.Z.); 2 National and Local Joint Engineering Research Center of Technical Fiber Composites for Safety and Health, Nantong University, Nantong 226019, China

**Keywords:** electrospun nanofibers, regenerated cellulose, ZIF-8, adsorption, tetracycline hydrochloride

## Abstract

The excessive utilization of antimicrobials in humans and animals has resulted in considerable environmental contamination, necessitating the development of high-performance antibiotic adsorption media. A significant challenge is the development of composite nanofibrous materials that are both beneficial and easy to fabricate, with the aim of improving adsorption capacity. Herein, a new kind of zeolitic imidazolate framework-8 (ZIF-8)-modified regenerated cellulose nanofibrous membrane (ZIF-8@RC NFM) was designed and fabricated by combining electrospinning and in situ surface modification technologies. Benefiting from its favorable surface wettability, enhanced tensile strength, interconnected porous structure, and relatively large specific surface area, the resulting ZIF-8@RC NFMs exhibit a relatively high adsorption capacity for tetracycline hydrochloride (TCH) of 105 mg g^−1^ within 3 h. Moreover, a Langmuir isotherm model and a pseudo-second-order model have been demonstrated to be more appropriate for the description of the TCH adsorption process of ZIF-8@RC-3 NFMs. Additionally, this composite fibrous material could keep a relatively stable adsorption capability under various ionic strengths. The successful fabrication of the novel ZIF-8@RC NFMs may shed light on the further development of wastewater adsorption treatment materials.

## 1. Introduction

Antibiotics are widely used in various fields, including the medical industry, agriculture, animal husbandry, and scientific research, as important antibacterial drugs. Among the many antibiotics, tetracycline hydrochloride (TCH), as a representative antibiotic with a broad-spectrum bactericide effect, has been extensively utilized in the aquaculture and animal husbandry industries [1,2]. However, the widespread use of antibiotics in humans and animals has caused serious contamination to soil and water, despite their bacteriostatic and bactericidal effects. This is because TCH is excreted into the underground sewer system through fecal matter, which is metabolized by the human body, livestock, and fish. Due to the low treatment performance of sewage treatment plants, these TCH pollutants usually seep into rivers or groundwater [3,4]. TCH-containing wastewater typically exhibits several problematic characteristics, including high biological toxicity, concentrated organic substances, strong antibacterial properties, and resistance to degradation. These characteristics can lead to the enhanced resistance of human intestinal bacteria and have a significant impact on the growth of bones and teeth [5,6]. Therefore, it is critical to focus on and make significant efforts to remove low levels of antibiotic contaminants from wastewater.

Currently, several methods are used for antibiotic wastewater treatment, including adsorption, membrane separation, ion exchange, oxidation and advanced oxidation, photocatalysis, and aerobic and anaerobic biological processes [7,8,9]. Among these approaches, the adsorption approaches have been extensively applied, benefitting from their easy manipulation, simple device usage, environmental safety, and high treatment efficiency [10]. To date, various adsorption materials have been developed to realize the effective removal of TCH pollutants from wastewater, including graphene, carbon nanotube, activated carbon, biochar, metal–organic frameworks (MOFs), and composite materials [11,12,13,14,15,16]. Further, polymetallic MOFs have emerged as a promising class of adsorbents due to a number of advantageous characteristics, including a large specific surface area, abundant active sites, a rich porous structure, excellent structural adjustability, and favorable compatibility with polymer matrices [5,17,18,19]. Among the series of MOFs, zeolitic imidazolate framework-8 (ZIF-8) has demonstrated considerable potential for application in wastewater processing, owing to its favorable thermal and chemical structural stability in neutral or weakly alkaline environments, good water stability, easy preparation, and adjustable porosity [20,21]. Taking advantage of these exceptional properties, ZIF-8-based MOFs have been widely developed and applied for the disposal of antibiotic-laden wastewater [22,23,24]; however, the currently synthesized ZIF-8 adsorbents are generally granular, which usually makes them difficult to completely separate from water and easily causes secondary pollution [23,25,26]. Accordingly, an effective approach to addressing this issue is to securely attach the ZIF-8 particles to the substrate, thereby enabling them to fully realize their adsorption potential while simultaneously ensuring their recyclability.

Electrospun nanofibers have demonstrated a number of outstanding characteristics since their development, such as having a large fiber aspect ratio, good fiber continuity, favorable structural controllability, high porosity, and being rich in species. These prominent advantages have enabled electrospun nanofibers to show broad application prospects in many fields, including tissue engineering, drug delivery, sensors, energy application, air filtration, catalytic degradation, etc. [27,28,29]. It should be noted that the natural interconnected pores created by electrospinning between the fibers are very conducive to fluid transport. Thus, the combination of ZIF-8 and electrospun nanofibers can fully exploit the structural advantages of ZIF-8 and effectively improve the defects in its application. At present, ZIF-8/nanofiber composites can be prepared by using blended spinning technology, surface modification approaches, and in situ growth methods [23,30,31,32]. In comparison, the use of in situ growth methods to immobilize ZIF-8 nanostructures onto the surface of nanofibrous membranes is considered to be one of the more effective means of synthesizing ZIF-8/nanofiber composites, due to the fact that this approach can effectively utilize the adsorption sites within these nanostructures fixed on the nanofiber surface. Electrospun polyacrylonitrile (PAN) nanofibers have been commonly used as substrates for the deposition of ZIF-8 nanoparticles and have exhibited superior adsorption performance [33]. However, the alkali swelling resistance of the PAN nanofiber substrate is relatively weak, which limits its application in the field of water treatment. Alternatively, silicon dioxide (SiO_2_) nanofibrous membranes have been employed as substrates for the growing of ZIF-8 nanoscrystals, whereas their mechanical performance still needs to be further improved [34]. Therefore, the current challenge is to select a nanofiber membrane with good hydrophilicity, excellent mechanical properties, and favorable acid and alkali resistance as a substrate, and use a facile method to attach ZIF-8 nanocrystals to its surface for the efficient treatment of antibiotics.

Electrospun regenerated cellulose nanofibrous membranes (RC NFMs), with the favorable properties of good hydrophilicity, strong mechanical strength, superior chemical stability, and abundant active hydroxyl groups, could be suitable substrates for firmly anchoring ZIF-8 nanoparticles [35,36]. Here, robust ZIF-8-modified regenerated cellulose nanofibrous membranes (ZIF-8@RC NFMs) were developed by combining electrospinning techniques and in situ surface modification approaches. To our knowledge, there has been no research on the design of such fiber composites. The morphological structure, chemical constitution, mechanical strength, surface wettability, TCH adsorption, and removal performance of the original nanofibrous substrate and the resulting composite ZIF-8@RC NFMs were systematically investigated. The results show that ZIF-8 nanocrystals are successfully attached to the nanofiber surface. Due to the effective loading of functional ZIF-8 nanoparticles, the favorable wettability and tensile strength, and the porous structure with open and interconnected channels, the ZIF-8@RC NFMs exhibit a relatively good TCH adsorption performance. The influence of ionic strength and pH value on the TCH adsorption performance of the resulting fibrous composites was investigated. The development of the novel ZIF-8@RC NFMs may provide a promising strategy for designing high-performance materials for wastewater treatment.

## 2. Results and Discussion

**Figure 1:**
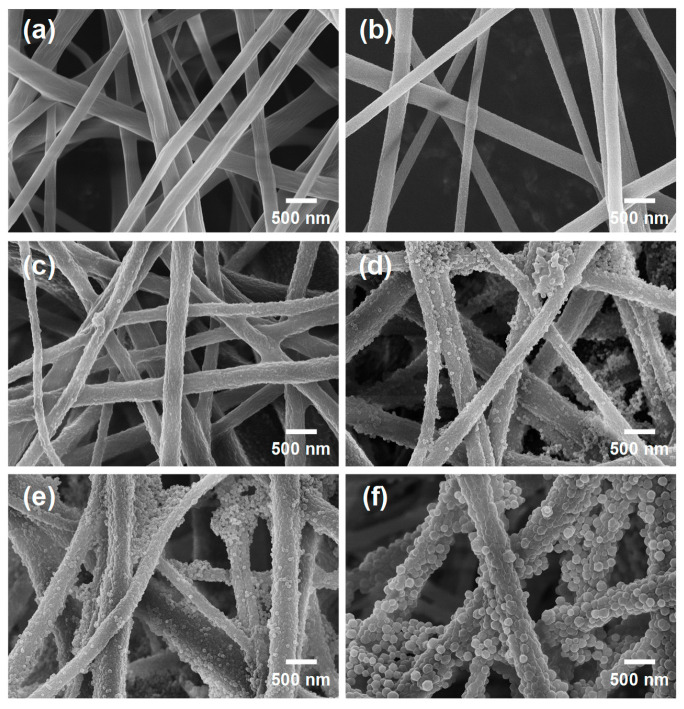
FE-SEM photographs of (**a**) CA, (**b**) RC, (**c**) ZIF-8@RC-1, (**d**) ZIF-8@RC-2, (**e**) ZIF-8@RC-3, and (**f**) ZIF-8@RC-4 NFMs.

The preparation process of the ZIF-8@RC NFMs mainly involved three steps. First, CA NFMs were fabricated by using an electrospinning technique. Then, the CA NFMs were treated with NaOH solution to obtain RC NFMs. Subsequently, ZIF-8 particles were decorated onto the RC nanofibers’ surfaces using in situ growth and deposition methods. The morphological structure of the CA NFMs, RC NFMs, and the resulting ZIF-8@RC NFMs was characterized by FE-SEM. As displayed in Figure 1a,b, both the CA and RC NFMs exhibited relatively smooth fiber surfaces. The statistical average diameter of the CA and RC nanofibers was 376 and 345 nm, respectively. The slight change in the average diameter could be attributed to the effect of the deacetylation reaction under an alkaline environment [37]. After the surface modification process, an obvious rough structure was observed on the surface of RC nanofibers. Significantly, a slight rough structure incubated with nanoparticles could be observed on the fiber surface of the ZIF-8@RC-1 NFMs (Figure 1c). After increasing the concentration of Zn(NO_3_)_2_ and 2-methylimidazole in the solution, obvious ZIF-8 nanoparticle-structured coating layers could be observed around the outside of the individual RC nanofibers or between the nanofibers (Figure 1d–f). With the increasing concentration of the modified substance, the roughness of the fiber surface and the size of the functional ZIF-8 nanoparticles gradually increased.

**Figure 2:**
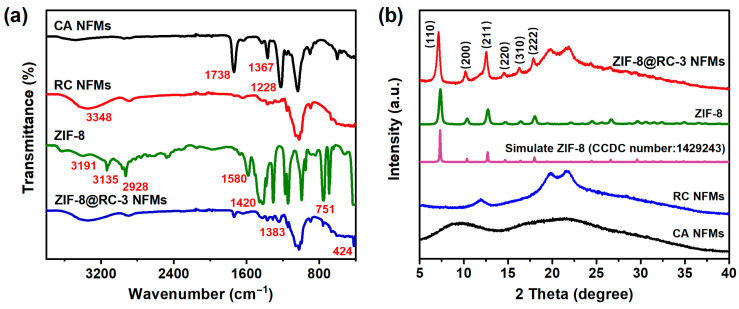
(**a**) FT-IR and (**b**) XRD spectra of the CA, RC, ZIF-8 particle, and representative ZIF-8@RC-3 NFMs.

To further illustrate the chemical structure of the targeted ZIF-8@RC NFMs, FT-IR, XRD, and SEM-EDS measurements of the corresponding samples were performed. As shown in Figure 2a, compared to the FT-IR spectra of raw CA NFMs, the significantly enhanced –O–H characteristic peak around 3348 cm^−1^, and the obvious decrease in the stretching vibrations of –C=O (1738 and 1228 cm^−1^) and –C–O–C (1367 cm^−1^), demonstrated the relatively complete deacetylation treatment of the CA NFMs and the successful preparation of the RC NFMs [37,38]. As evidenced by the FT-IR spectrum of the synthesized ZIF-8 particles, the characteristic peaks at 1580, 2928, 3135, and 3191 cm^−1^ were assigned to C=N, aliphatic C–H, aromatic C–H stretching vibrations, and N–H stretching vibrations of the imidazole rings in ZIF-8 particles [34,39,40]. The adsorption band at 1420 cm^−1^ was related to the C–N stretching vibration of the imidazolate ring. The characteristic peaks between 900 and 1350 cm^−1^ were allocated to the imidazolate ring in-plane bending [41]. The band at 752 cm^−1^ belonged to the bending vibration of the imidazole ring, and the adsorption band at 424 cm^−1^ corresponded to the Zn–N stretching [42]. The aforementioned characteristic peaks exhibited a high degree of correlation with those reported in previous research works, tentatively indicating the successful synthesis of ZIF-8 nanoparticles. In addition, these characteristic peaks were observed for ZIF-8@RC-3 NFMs, demonstrating the effective anchoring of ZIF-8 crystal structures on RC NFMs.

In addition, the chemical structure of these relevant nanofibrous materials was further investigated by XRD. The XRD patterns of the CA NFMs, RC NFMs, ZIF-8 particle, and representative ZIF-8@RC-3 NFMs are presented in Figure 2b. In the case of CA NFMs, almost no crystallization peak was observed. However, following alkali treatment, the synthesized RC NFMs exhibited a pronounced diffraction crystal peak at 11.88°, 19.82°, and 21.6°. The observed peaks were consistent with the standard spectrum of cellulose (JCPDS No.: 03-0226), thereby confirming the successful synthesis of cellulose II [35,43]. The XRD spectra of the prepared ZIF-8 particles fitted well with the simulated ZIF-8 spectrum (CCDC number: 1429243), confirming the successful synthesis of ZIF-8. It is also noteworthy that the newly appeared diffraction peaks could be observed from the ZIF-8@RC-3 NFMs at 7.14°, 10.18°, 12.54°, 14.54°, and 16.3°, corresponding to the (110), (200), (211), (220), (310), and (222) diffraction planes of ZIF-8 [44,45,46], respectively.

**Figure 3:**
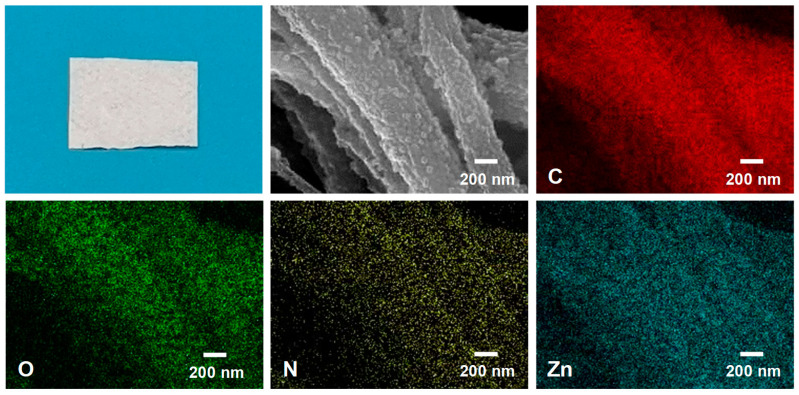
FE-SEM-EDS elemental mapping of the ZIF-8@RC-3 NFMs for C, O, N, and Zn.

Moreover, the physical characteristics and distribution of the target elements in the composites are illustrated in Figure 3. The representative sample of ZIF-8@RC-3 NFMs exhibited an almost pure white appearance. The EDS mapping images showed that, in addition to the C and O elements found in the RC NFMs, there were also distinct signal peaks for N and Zn on the surface of the ZIF-8@RC-3 NFMs. The preceding experimental results demonstrated that ZIF-8 particles were effectively deposited onto the RC NFM substrate.

**Figure 4:**
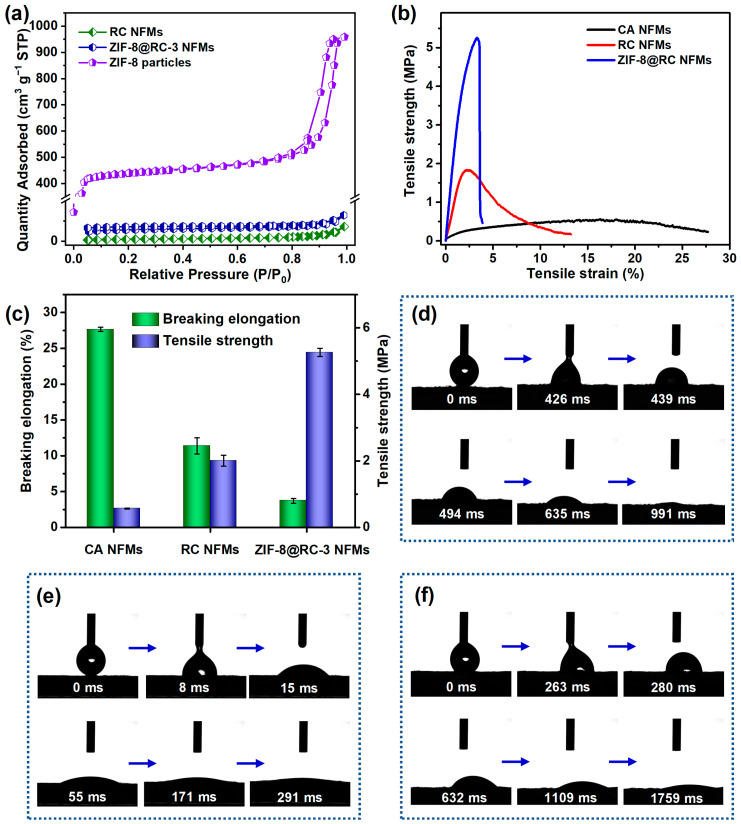
(**a**) Nitrogen adsorption–desorption isotherms of RC, ZIF-8@RC-3 NFMs, and ZIF-8 particles. (**b**) The representative tensile curves and (**c**) the corresponding elongation at break and tensile strength of the relevant membranes. The recording photos of the dynamic permeation process of water droplets on the surface of (**d**) CA NFMs, (**e**) RC NFMs, and (**f**) ZIF-8@RC-3 NFMs.

As for the adsorbent, the pore structure has a certain influence on its application performance. Therefore, the nitrogen adsorption–desorption isotherms of these membranes before and after ZIF-8 modification, as well as the synthesized ZIF-8 particles, were subjected to testing. As shown in Figure 4a, the adsorption of nitrogen molecules on the pristine RC NFMs followed a type III adsorption isotherm, indicating a solid and non-porous structure on the fiber surface. Conversely, the ZIF-8@RC-3 NFMs showed a rapid increase in adsorption at low pressures, characterized by a type II isotherm profile. For the ZIF-8 particles obtained by a similar synthetic route, the nitrogen adsorption–desorption isotherm exhibited the characteristics of the type IV isotherm, including single and multilayer nitrogen molecular adsorption and capillary condensation phenomena [47]. The resulting BET SSAs of the RC NFMs, ZIF-8@RC-3 NFMs, and ZIF-8 particles were 3.47, 18.01, and 1748.13 m^2^ g^−1^ (Table S1), respectively. In addition, the total pore volumes of these materials were 0.011, 0.020, and 1.486 cm^3^ g^−1^, respectively. Compared with the original RC NFMs, the introduction of ZIF-8 particles into ZIF-8@RC-3 NFMs could enable an increase in the BET SSA and the total pore volume. However, the BET SSA of the MOF particles formed in situ on the fiber surface was significantly reduced compared to the MOF particles. This phenomenon has also been observed in other works in the literature [48,49]. The observed reduction in the BET SSA may be due to the fact that during the in situ growth of MOF particles within the membranes, these particles continue to extend outward along the seed crystals attached to the fiber surface, forming a multi-layer stacking of particles, thereby reducing the BET SSA.

The mechanical properties of the materials are critical for long-term use in practical applications, thus the tensile mechanics of the resulting fibrous membranes are being systematically investigated. Apparently, as can be seen from the representative tensile stress–strain curves of the CA, RC, and ZIF-8@RC-3 NFMs (Figure 4b), the tensile stress of the CA NFMs increased slowly and with low amplitude during the tensile test. The RC NFMs exhibited relatively enhanced tensile stress and decreased elongation at break. However, the tensile strength of ZIF-8@RC-3 NFMs increased rapidly and largely to the maximum amount, and then fractured at a relatively small tensile strain. The analysis results of multiple tensile tests demonstrated that the CA, RC, and ZIF-8@RC-3 NFMs exhibited a gradually increasing tensile strength average value of 0.56, 2.0, and 5.26 MPa, with a decreasing elongation at break of 27.66%, 11.38%, and 3.72%, respectively (Figure 4c). The different mechanical properties of these membranes could be attributed to their different microstructures. As can be seen from the FE-SEM characterization results, the CA nanofibers were randomly and disorderly distributed in the membranes with no effective bonding or adhesion among the nanofibers, and thus were prone to slip during the tensile measurement process. Therefore, the CA NFMs presented a relatively lower tensile strength and higher breaking elongation at break. After treatment with NaOH solution and drying effects, the relatively strong hydrogen bonds were formed among the RC nanofibers due to their high hydroxyl content, resulting in improved tensile mechanical properties [50]. After the functional surface modification process, an obvious bonding interaction assembled by ZIF-8 nanoparticles could be observed among the nanofibers within representative ZIF-8@RC-3 NFMs. As a result, the tensile mechanical performance of the composite membrane was greatly enhanced, which was beneficial to the wastewater treatment application performance of the composite ZIF-8@RC NFMs.

Figure 4d–f show the dynamic water wetting process of these relevant membranous materials. During the dynamic wettability test process, the water droplet detached from the test needle and adhered to the CA NFMs at about 439 ms, and the CA NFMs exhibited a relatively large initial water contact angle of close to 90°. The water droplet infiltrated almost completely into the fiber membrane at 991 ms, illustrating the relatively poor water wettability of the original CA NFMs. Because of the abundance of -OH on the fiber surface, the RC NFMs exhibited a relatively small initial water contact angle and a shorter complete wetting time of 291 ms, indicating the improved surface wettability. However, the complete wetting time of the water droplets of the representative ZIF-8@RC-3 NFMs was up to 1759 ms, demonstrating that the fibrous materials had reduced hydrophilic surface wettability. These results could be ascribed to the rough surface structure assembled by the large number of formed ZIF-8 nanoparticles [51,52]. According to the Cassie–Baxter model, when the water droplet is dropped on the rough surface of the ZIF-8@RC-3 NFMs, the spherical shape of the water droplet can be maintained over a specific duration due to the effect of the surface tension of water [53]. With the prolongation of the contact time, the water droplet could slowly infiltrate into the composite fibrous membrane due to its porous fibrous structure, which still exhibits a hydrophilic property.

**Figure 5:**
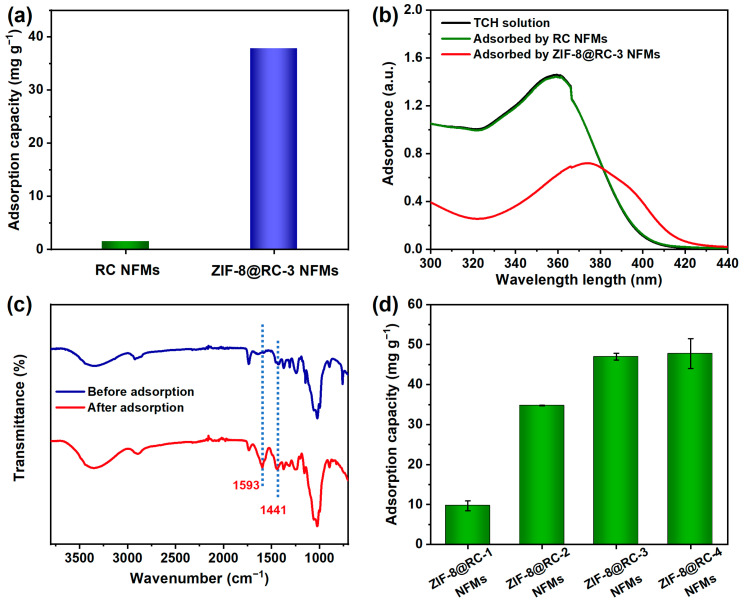
(**a**) Comparison of TCH adsorption capacity of RC NFMs and ZIF-8@RC-3 NFMs. (**b**) UV-vis spectra of the TCH solution before and after adsorption by the relevant samples. (**c**) FT-IR spectra of ZIF-8@RC-3 NFMs before and after TCH adsorption. (**d**) TCH adsorption capacities of these ZIF-8@RC NFMs prepared by using different concentrations of reagents.

Herein, TCH was taken as the main template with which to test the adsorption performance of the resulting ZIF-8@RC NFMs for antibiotic contaminants. The UV-vis absorption spectra of the initial TCH solution (40 mg L^−1^) and the representative solutions after the adsorption treatment for 1 h are depicted in the inset of Figure 5a. As compared with the initial TCH solution, almost no obvious change could be observed from the solution after adsorption by the RC NFMs. However, the absorbance value of the characteristic peak of the TCH was significantly reduced after adsorption by the ZIF-8@RC-3 NFMs. The calculated TCH adsorption amount of the representative ZIF-8@RC-3 NFMs was significantly higher than the RC NFMs (Figure 5b). The chemical structure of ZIF-8@RC-3 NFMs before and after TCH adsorption was characterized using FT-IR. Obviously, after the adsorption process, the feature peaks at 1593 and 1441 cm^−1^ could be attributed to the TCH molecules adsorbed on the ZIF-8@RC-3 NFMs (Figure 5c), demonstrating the effective capture and removal of TCH by ZIF-8@RC-3 NFMs [2,54].

Moreover, we further tested and analyzed the TCH adsorption capacities of these ZIF-8@RC NFMs prepared by using different concentrations of Zn(NO_3_)_2_ and 2-methylimidazole solutions. As can be seen from Figure 5d, under the same adsorption conditions of an initial TCH of 40 mg L^−1^ and an adsorption time of 4 h, the ZIF-8@RC-1 NFMs exhibited a relatively lower adsorption of 9.7 mg g^−1^, whereas, after increasing the concentrations of Zn(NO_3_)_2_ and 2-methylimidazole in the modification solution, the TCH adsorption capacity of ZIF-8@RC-3 NFMs could reach up to 47 mg g^−1^, which was almost close to the adsorption performance of the ZIF-8@RC-4 NFMs. This phenomenon could be ascribed to the increasing loading of functional ZIF-8 particles on the RC NFMs, resulting in the improvement of the TCH removal performance.

**Figure 6:**
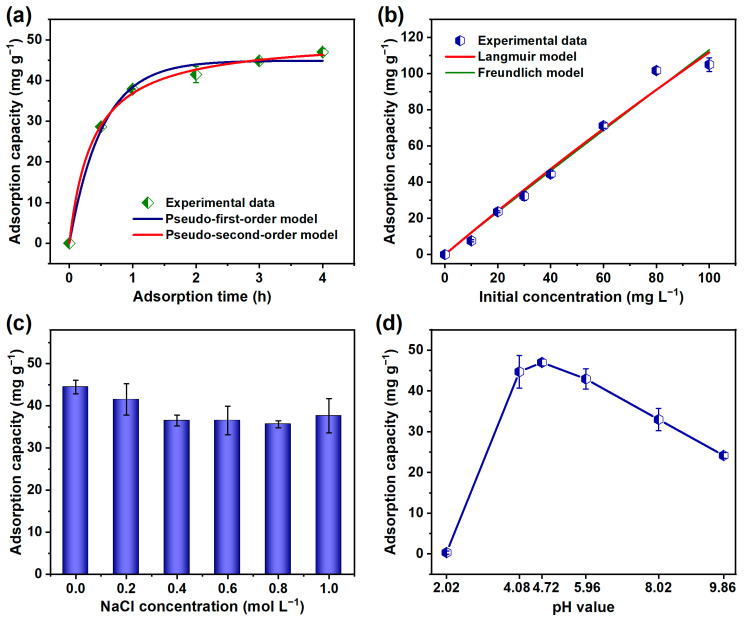
(**a**) Adsorption kinetics and (**b**) adsorption isotherms, as well as the corresponding fitting curves of TCH on the ZIF-8@RC-3 NFMs. Influences of (**c**) ionic strength and (**d**) pH on the TCH adsorption performance of the resulting ZIF-8@RC-3 NFMs.

We further investigated the impact of adsorption time on the TCH removal capability of the resulting materials through taking ZIF-8@RC-3 NFMs as a representative sample. As exhibited in Figure 6a, the TCH adsorption amount of the ZIF-8@RC-3 NFMs increased relatively rapidly within the first 1 h, then increased slightly and tended to reach adsorption saturation along with the adsorption time of up to 4 h. The relationship between the adsorption capacity of the ZIF-8@RC-3 NFMs and the TCH concentration was investigated by introducing pseudo-first-order and pseudo-second-order kinetics (Equations (S1) and (S2)) [5,24]. As can be seen from the resulting fitting curves and corresponding fitting parameters (Table 1), the TCH adsorption process of the ZIF-8@RC-3 NFMs fitted well with the pseudo-second-order kinetics due to the relatively high correlation coefficients (R^2^ = 0.998), demonstrating that the chemical adsorption interaction mainly determines and dominates the TCH adsorption process on the ZIF-8@RC-3 NFMs [22,32,55]. The resulting equilibrium TCH adsorption capability of the ZIF-8@RC-3 NFMs calculated using the pseudo-second-order kinetics was 50.66 mg g^−1^, which was approximately in line with the experimental adsorption capacity of 47 mg g^−1^.

Furthermore, we investigated the isothermal TCH adsorption process of ZIF-8@RC-3 NFMs by measuring the TCH adsorption capacity of the membranous materials under different initial TCH concentrations ranging from 0 to 100 mg L^−1^. Significantly, as the TCH concentration rose from 0 to 80 mg L^−1^, the adsorption capacity of the ZIF-8@RC-3 NFMs showed a nearly linear increase, with a high fitting coefficient of 0.993 (Figure 6b). The adsorption capacity tended to be balanced when the initial TCH concentration was further increased to 100 mg L^−1^. The TCH adsorption isotherms of the ZIF-8@RC-3 NFMs were further evaluated by introducing the Langmuir and Freundlich models (Equations (S3) and (S4)) [32,56], respectively. As can be seen from the fitting results (Table 2), the Langmuir model exhibited a slightly higher correlation coefficient compared to the Freundlich model, suggesting that the TCH adsorption by ZIF-8@RC-3 NFMs is primarily consistent with heterogeneous single-layer adsorption [57]. The Langmuir model estimated the maximum TCH adsorption capacity of the ZIF-8@RC-3 NFMs to be 1230.18 mg g^−1^, highlighting their significant potential for TCH removal applications.

Moreover, the effects of ionic strength and pH on the TCH removal performance of the ZIF-8@RC-3 NFMs were further investigated. As shown in Figure 6c, the TCH adsorption amount of the ZIF-8@RC-3 NFMs slightly decreased with the increase in NaCl concentration from 0 to 0.4 mol L^−1^, and the composite membranes maintained an almost unchanged TCH adsorption capability with the further increase in ionic strength. This phenomenon could be ascribed to the chemical adsorption interaction between TCH and the ZIF-8@RC-3 NFMs, which was largely unaffected by ionic strength, leading to the conclusion that ionic strength has a minimal effect on the performance of composite materials in practical applications [58]. The TCH adsorption capacities of the representative ZIF-8@RC-3 NFMs at different pH values are displayed in Figure 6d. As the pH increased from 2.02 to 4.08, the TCH adsorption capacity of the ZIF-8@RC-3 NFMs significantly increased from 0.31 to 44.71 mg g^−1^. The ZIF-8@RC-3 NFMs maintained a relatively stable TCH adsorption capacity as the pH increased from 4.08 to 5.96, whereas the TCH adsorption capacity decreased to 24.17 mg g^−1^ with the further increase in the pH value of TCH solution. The optimum TCH adsorption amount of the ZIF-8@RC-3 NFMs was obtained at the pH of the original TCH solution (40 mg L^−1^), which was 4.72. The experimental results were consistent with previous research [32,55]. This result could be ascribed to the synergistic effects of pH on the surface charge of ZIF-8-based composited materials and TCH, the coordinate bonds, and the π-π interactions [11,59,60].

According to the experiment results and the literature reports [61], the adsorption mechanism of the composite fiber membrane was analyzed as follows: the metal active sites on the ZIF-8 particles form a coordination bond with the highly electronegative atoms in the TCH molecule, which promotes the adsorption of TCH on the surface of the ZIF-8@RC-3 NFMs [22,58]. Furthermore, the hydrogen and π-π bonding, as well as the electrostatic interaction between ZIF-8 particles and TCH molecules, will affect the adsorption effect of TCH molecules on the surface of ZIF-8@RC-3 NFMs [22,33,61]. Additionally, the TCH adsorption performance of the ZIF-8@RC NFMs was evaluated in comparison to other TCH adsorbents that have been documented in the literature. As presented in Table S2, the TCH adsorption performance of the ZIF-8@RC NFMs outperformed most other TCH adsorbents reported in the literature, demonstrating the competitive TCH removal efficiency of these materials.

## 3. Materials and Methods

### 3.1. Materials

Cellulose acetate (CA, molecular weight of 30,000, acetyl content of 39.8%, and hydroxyl content of 3.5%) and 2-methylimidazole were provided by Shanghai Aladdin Biochemical Technology Co., Ltd. (Shanghai, China). Zinc nitrate hexahydrate (Zn(NO_3_)_2_·6H_2_O) and acetone were supported by Sinopharm Chemical Reagent Co., Ltd. (Shanghai, China). N,N-dimethylacetamide (DMAc) was supplied by Shanghai Macklin Inc. (Shanghai, China). Methanol was obtained from Shanghai Zhenxing No.1 Chemical Plant (Shanghai, China). Tetracycline hydrochloride (TCH) was purchased from Dalian Meilun Biotech Co., Ltd. (Dalian, China). Sodium chloride (NaCl) was provided by Taicang Zhoushi Chemical Co., Ltd. (Taicang, China). All chemicals used in this study were employed in their as-received state.

### 3.2. Preparation of the ZIF-8@RC NFMs

First, a cellulose acetate solution with a concentration of 17.5% was prepared by dissolving cellulose acetate powder in a mixed solvent of DMAc and acetone (mass ratio of 1:2), stirring it at room temperature (about 25 °C) for 12 h. The prepared cellulose acetate solution was conducted by an electrospinning process with the parameters of the direct current voltage of 25 kV, the perfusion rate of 0.5 mL h^−1^, the spinning distance of 15 cm, and the roller speed of 50 rpm. The resulting cellulose acetate nanofibrous membranes (CA NFMs) were immersed into a NaOH aqueous solution with a concentration of 0.1 mol L^−1^ and treated at 60 °C for 30 min. Then, the fibrous membranes were thoroughly washed until neutral with plenty of deionized water and dried in an oven to obtain RC NFMs.

The ZIF-8@RC NFMs were synthesized by in situ deposition of ZIF-8 nanostructures on the fiber surface of the RC NFMs. Zinc nitrate hexahydrate solution with concentrations of 0.009, 0.018, 0.036, and 0.071 mol L^−1^, as well as 2-methylimidazole solution with concentrations of 0.23, 0.45, 0.91, and 1.81 mol L^−1^, were prepared using methanol as the solvent, respectively. During the modification process, 0.02 g of RC NFMs was incubated in a conical flask containing Zn(NO_3_)_2_ solution and gently shaken so that the membranes were completely immersed and in contact with the Zn(NO_3_)_2_ solution. Then, a certain volume of 2-methylimidazole solution was introduced into the conical bottle. After gentle shaking, the mixed solution was allowed to stand for a period of time to realize the in situ synthesis and deposition of ZIF-8 nanostructures on the surface of regenerated cellulose nanofibers. Subsequently, the modified membranes were taken out using tweezers and transferred to a new beaker, and then washed thoroughly by using anhydrous ethanol. Finally, the ZIF-8-modified RC NFMs (ZIF-8@RC NFMs) were successfully prepared after the drying process. A series of composite nanofibrous membranes were synthesized using different concentrations of Zn(NO_3_)_2_ and 2-methylimidazole solutions, and the corresponding samples were labeled as presented in Table 3.

### 3.3. Measurement of the TCH Adsorption Performance

The static TCH adsorption capability of the relevant membranous materials was determined by bath adsorption experiments. A certain amount of TCH aqueous solution was injected into a brown reagent bottle, and a certain weight of ZIF-8@RC NFMs was added into the bottle. The subsequent adsorption process was performed on a magnetic stirrer at a gentle agitating rate. After centrifugation for 10 min at 4000 rpm, the absorbance of the collected supernatant was characterized through ultraviolet–visible (UV-vis) spectrophotometry. Finally, the TCH adsorption capacity of the sample can be calculated based on the change in absorbance at the characteristic peak, as well as the volume and mass of TCH and the ZIF-8@RC NFMs used in the adsorption experiment, respectively. To investigate the effects of TCH concentration, adsorption time, and ionic strength on the performance of ZIF-8@RC NFMs, the adsorption capacity was systematically measured under different initial TCH concentrations (0 to 100 mg L^−1^), different times (0 to 4 h), and different ionic strengths (0 to 1 mol L^−1^ NaCl). Furthermore, to explore the influence of pH value on the adsorption capacity of ZIF-8@RC NFMs, a TCH solution with different pH levels (2.02, 4.08, 4.72, 5.96, 8.02, and 9.86) was prepared using 0.1 mol L^−1^ sodium hydroxide and hydrochloric acid.

### 3.4. Instruments and Characterizations

The morphological structures of the relevant membranes, including CA NFMs, RC NFMs, and the resulting ZIF-8@RC NFMs, were featured using a field-emission scanning electron microscope (FE-SEM) (Gemini SEM 300, Carl Zeiss, Oberkochen, Germany). The chemical structure of the relevant membranous materials was measured through Fourier transform infrared (FT-IR) spectroscopy (Nicolet 5700, Thermo Fisher Nicolet, Waltham, MA, USA). The crystal structure was characterized using X-ray diffraction (XRD, Ultima IV, Rigaku, Tokyo, Japan). The pore structures of the synthesized materials were recorded utilizing an ASAP 2020 automatic physical adsorption analyzer (Micromeritics, Norcross, GA, USA), including the measurements of the Brunauer–Emmett–Teller specific surface area (BET SSA), the total pore volume, and the mean pore size. The dynamic surface wettability and tensile strength of these membranous materials were measured using an SDC-350 contact angle meter (Dongguan SINDIN Precision Instrument Co., Ltd., Dongguan, China) and an XQ-2 tensile tester (Shanghai New Fiber Instrument Co., Ltd., Shanghai, China). The absorbance curves of the TCH solution before and after the adsorption process were recorded using a TU-1810 UV-vis spectrophotometer (Beijing Puxi General Instrument Co., Ltd., Beijing, China).

## 4. Conclusions

In conclusion, this work demonstrated a facile fabrication strategy for ZIF-8 composite nanofibrous membranes through the combination of electrospinning and in situ surface deposition modification technologies. The morphological structure of ZIF-8@RC NFMs was tailored by regulating the loading amount of the modification reagents. The mechanical performance of the composite membranes was significantly improved after the introduction of ZIF-8. Profiting from the effective adsorption points of the functional ZIF-8 nanoparticles, favorable surface wettability, interconnected porous structure, and enhanced mechanical strength, the ZIF-8@RC NFMs exhibited a relatively high TCH adsorption capacity of 105 mg g^−1^ within 3 h. Isothermal and kinetic adsorption studies illustrated that the TCH adsorption process of the resulting composite membranes matched well with Langmuir model and pseudo-first-order kinetics. The theoretical adsorption amount was calculated to be approximately 1230.18 mg g^−1^, highlighting the significant potential for the application of the obtained composite membranous adsorbents. This work is expected to provide a reference for the design and fabrication of functional nanofiber/MOF composites with broad application prospects in the removal of antibiotic contaminants.

## Figures and Tables

**Table 1:** The fitting kinetic parameters of TCH adsorption on ZIF-8@RC-3 NFMs.

Pseudo-First-Order Kinetic Model			Pseudo-Second-Order Kinetic Model		
*q_e_* (mg g^−1^)	*k*_1_ (h^−1^)	*R* ^2^	*q_e_* (mg g^−1^)	*k*_2_ (g mg^−1^ h^−1^)	*R* ^2^
44.87	1.928	0.991	50.66	0.053	0.998

**Table 2:** Fitting parameters of the Langmuir and Freundlich models for TCH adsorption.

Langmuir Model			Freundlich Model		
*q_m_* (mg g^−1^)	*K_L_* (L mg^−1^)	*R* ^2^	*K_F_*	*1/n*	*R* ^2^
1230.18	0.001	0.979	16.11	1.408	0.978

**Table 3:** Preparation parameters of the different types of ZIF-8@RC NFMs.

Sample Name	Concentration of Zn(NO_3_)_2_ (mol L^−1^)	Concentration of 2-Methylimidazole (mol L^−1^)
ZIF-8@RC-1 NFMs	0.009	0.23
ZIF-8@RC-2 NFMs	0.018	0.45
ZIF-8@RC-3 NFMs	0.036	0.91
ZIF-8@RC-4 NFMs	0.071	1.81

## Data Availability

The data used to support the findings of this study are available from the corresponding author on request.

## Supplementary Materials

The following supporting information can be downloaded at https://www.mdpi.com/article/10.3390/molecules29174146/s1: Supplementary methods: kinetic and isothermal adsorption process analysis; Table S1: The pore structure parameters of the resultant materials; Table S2: Comparison of TCH adsorption capacity with different adsorbents; Supplementary references [62,63,64,65,66,67,68,69].
