# Supplementary material for: Facile Fabrication of Zeolitic Imidazolate Framework-8@Regenerated Cellulose Nanofibrous Membranes for Effective Adsorption of Tetracycline Hydrochloride
Source: Molecules. 2024 Aug 31;29(17):4146. doi: 10.3390/molecules29174146 (PMC11397351; doi:10.3390/molecules29174146)
Supplement: Supplementary file 1 [file molecules-29-04146-s001.zip › molecules-3129836-supplementary.pdf]

molecules MDPI

Supplementary Information

Facile Fabrication of Zeolitic Imidazolate Framework-8@Regenerated Cellulose Nano-fibrous Membranes for Effective Adsorption of Tetracycline Hydrochloride

Zhirong Wang [1], Qiuxia Fu [1,2,*], Dandan Xie [1], Fujie Wang [1], Guangyu Zhang [1,2] and Haoru Shan [1,2,*]

[1] School of Textile and Clothing, Nantong University, Nantong 226019, China
[2] National and Local Joint Engineering Research Center of Technical Fiber Composites for Safety and Health, Nantong University, Nantong 226019, China
* Correspondence: fuqx@ntu.edu.cn (Q.F.); hrshan@ntu.edu.cn (H.S.)

Supplementary Methods

Adsorption kinetic and isotherm studies of ZIF-8@RC-3 NFMs

TCH adsorption kinetics of the representative ZIF-8@RC-3 NFMs were simulated by the pseudo-first and second-order kinitics models, which are respectively presented by equation S1 and S2 [1,2]:

$$\ln(q_e - q_t) = \ln q_e - K_1 t \qquad \text{(Eq. S1)}$$

$$q_t = \frac{K_2 {q_e}^2 t}{1 + K_2 q_e t} \qquad \text{(Eq. S2)}$$

where q_t (mg g^{-1}) and q_e (mg g^{-1}) represent the equilibrium TCH adsorption amounts of ZIF-8@RC-3 NFMs at a given asorption time (t), respectively. K_1 and K_2 indicate the corresponding rate constants. The isothermal TCH adsorption of ZIF-8@RC-3 NFMs was systematically evaluated by introducing the Langmuir and Freundlich models, which are represented the following equations [1,2], respectively:

$$q_m = q_e \frac{(1 + K_L C_e)}{K_L C_e} \qquad \text{(Eq. S3)}$$

$$\lg q_e = \frac{\lg C_e}{n} + \lg K_F \quad \text{(Eq. S4)}$$

where q_m and q_e denote the maximum TCH adsorption capacity and adsorption amount of the ZIF-8@RC-3 NFMs under different TCH initial concentrations (C_e, mg L^{-1}), K_L represents the Langmuir constant, K_F and n remark the Freundlich capacity constant and adsorption intensity constant, respectively.

Table S1. The pore structure parameters of the resultant materials.

Sample name	BET surface area (m^2 g^{-1})	Total pore volume (cm^3 g^{-1})	Average pore size (nm)
RC NFMs	3.47	0.011	12.8
ZIF-8@RC-3 NFMs	18.01	0.020	4.4
ZIF-8 particles	1748.13	1.486	3.4

Table S2. Comparison of TCH adsorption capacity with different adsorbents.

Adsorbent	Initial concentration （mg L^{-1}）	Adsorption time	Q_e (mg g^{-1})	Ref.
MSW-MMT	20	/	8.39	3
ZIF-8/PDA/PAN fibers	50	72 h	76.27	4
AF-$MnFe_2O_4$-ZIF-67	~48	40 min	43.87	5
OBCC	100	24 h	30.72	6
MPSNMs	40	4 h	22.16	7
Ag/Ni-TiO_2	50	150 min	23.4	8
AC-ZrO_2/CeO_2 NCs	~20	1 h	26.75	9
ZIF-8/PANNM	400	25 h	255.92	10
ZIF-8@RC NFMs	40	3 h	47	This work
ZIF-8@RC NFMs	100	3 h	105	This work

Supplementary References:

1. Jian, N.; Dai, Y.; Wang, Y.; Qi, F.; Li, S.; Wu, Y. Preparation of polydopamine nanofibers mat as a recyclable and efficient adsorbent for simultaneous adsorption of multiple tetracyclines in water. *J. Clean. Prod.* **2021**, *320*, 128875.
2. Fu, Q.; Xie, D.; Ge, J.; Zhang, W.; Shan, H. Negatively Charged Composite Nanofibrous Hydrogel Membranes for High-Performance Protein Adsorption. *Nanomaterials* **2022,** *12,* 3500.
3. Premarathna, K.; Rajapaksha, A.; Adassoriya, N.; Sarkar, B.; Sirimuthu, N.; Cooray, A.; Ok, Y.; Vithanage, M. Clay-biochar composites for sorptive removal of tetracycline antibiotic in aqueous media. *J. Environ. Manage.* **2019**, *238*, 315–322.
4. Chao, S.; Li, X.; Li, Y.; Wang, Y.; Wang, C. Preparation of polydopamine-modified zeolitic imidazolate framework-8 functionalized electrospun fibers for efficient removal of tetracycline. *J. Colloid Interf. Sci.* **2019**, *552*, 506-516.
5. Kori, A.; Ramavandi, B.; Mahmoodi, S.; Javanmardi F. Magnetization and ZIF-67 modification of Aspergillus flavus biomass for tetracycline removal from aqueous solutions: A stable and efficient composite. *Environ. Res.* **2024**, *252*, 118931.
6. Zhang, K.; Zhang, L.; Dong, X.; Zhao, Y.; Li, F.; Cen, Q. Efficient adsorption of tetracycline hydrochloride on biochar-ceramsite composite: Optimization of response surface methodology and investigation of adsorption mechanism. *Mater. Today Sustain.* **2023**, *24*, 100525.
7. Shan, H.; Si, Y.; Yu, J.; Ding, B. Facile access to highly flexible and mesoporous structured silica fibrous membranes for tetracyclines removal. *Chem. Eng. J.* **2021**, *417*, 129211.
8. Ma, S.; Qin, Y.; Sun, K.; Ahmed, J.; Tian, W.; Ma, Z. Round-the-Clock Adsorption–Degradation of Tetracycline Hydrochloride by Ag/Ni-TiO_2. *Materials* **2024**, *17*, 2930.
9. Zhang, X.; Sathiyaseelan, A.; Zhang, L.; Lu, Y.; Jin, T.; Wang, M. Zirconium and cerium dioxide fabricated activated carbon-based nanocomposites for enhanced adsorption and photocatalytic removal of methylene blue and tetracycline hydrochloride. *Environ. Res*. **2024**, *261*, 119720.
10. Li, X.; Shao, H.; Ma, Q.; Yu, W.; Dong, X. Self-supporting flexible metal-organic framework-based electrospun nanofibers membrane for efficient removal of tetracycline from aqueous solutions. *J. Solid State Chem.* **2022,** *312*, 123233.
